# Fentanyl in an Infant: Taking Our Breath Away

**DOI:** 10.7759/cureus.39216

**Published:** 2023-05-19

**Authors:** Ivan Ivanov, Emily Weber, Eugene Javorsky

**Affiliations:** 1 Emergency Department, New York City (NYC) Health + Hospitals/South Brooklyn Health, Brooklyn, USA; 2 Pediatric Emergency Department, New York City (NYC) Health + Hospitals/South Brooklyn Health, Brooklyn, USA

**Keywords:** respiratory failure, toxicology, child abuse, cocaine, fentanyl, naloxone, opioid abuse, pediatric ingestion

## Abstract

Pediatric respiratory failure carries a wide differential diagnosis. Toxic ingestion should remain on the differential even at very young ages. There have been increasing reports of fentanyl overdoses among adults; however, this should be considered for accidental pediatric ingestion, especially considering its high potential for mortality.

A nine-month-old female presented to the pediatric emergency department with respiratory failure. The patient was noted to be bradypneic with miotic pupils, and therefore, naloxone was given intravenously (IV) with a positive response. The patient required numerous boluses of intravenous naloxone, which ultimately saved her from intubation. The patient’s laboratory results were later positive for fentanyl and cocaine.

Fentanyl ingestion has a high mortality rate, especially in pediatrics. With increasing fentanyl use, there is a potential for exposure due to not only child abuse and intentional toxicity but also exploratory ingestions.

## Introduction

Pediatric acute respiratory failure has a wide differential diagnosis, especially in infancy [1]. Most common causes of acute respiratory failure vary; however, infectious causes of the upper and lower respiratory system remain the most common respiratory causes, while non-respiratory etiologies include cardiogenic pathology, sepsis, and inborn errors of metabolism [1]. Pediatric ingestions do remain an important part of the differential diagnosis of respiratory failure [2]. Unfortunately, it is often difficult to obtain a comprehensive history and elicit a history of ingestion in the emergent stabilization of pediatric respiratory arrest [2]. One important ingestion to consider is opioid ingestion. Per Calello and Henretig [3], opioid ingestion affects mu receptors in the respiratory centers of the brainstem, which causes apnea and can lead to respiratory failure. This is especially true in a pediatric patient with an increased metabolic rate [3]. Using webPOISONCONTROL, an online resource showing statistics of overdoses reported to poison control centers, it was shown that there were 25 opioid overdoses in the age group under one year from January 2020 to August 2022, but with zero reported deaths [4]. Outside of what is reported to poison control, however, multiple case reports have shown that mortality with known fentanyl ingestion can be as high as 50% [5]. These cases were in the typical age group of pediatric ingestions of 2-4 years old. Other studies have shown that 3% of pediatric intensive care unit (PICU) admissions due to toxic ingestions have been due to opioid ingestion [6].

We present a case of a somnolent nine-month-old female who was bradypneic to six breaths a minute, in which a broad differential diagnosis was considered and ultimately a thorough physical examination showed findings compatible with opioid-induced respiratory failure, specifically miotic pupils. Some research has shown that the sensitivity of miotic pupils in opioid overdose can be as high as 91% [7]. Although it is well known that not all opioids cause miosis, it was used as a diagnostic aid in our specific case. This case highlights the importance of keeping a wide differential diagnosis in pediatric respiratory arrest and strongly considering toxic ingestions.

## Case presentation

A nine-month-old female born full-term via C-section with no medical history and a negative neonatal screen presented to the pediatric emergency department due to altered mental status and respiratory failure. The patient was found by her mother in her home, where both of the patient’s parents reside, “blue” and unresponsive. Emergency medical services (EMS) were called, and the patient was noted to be in respiratory failure and cyanotic. Bag valve mask (BVM) oxygenation was initiated, and a notification was called to the pediatric emergency department for a pediatric cardiac/pulmonary arrest. The patient arrived, cyanotic, and was ventilated with BVM by EMS personnel. The airway showed some secretions, and breath sounds were auscultated bilaterally. Pulses were immediately palpated. The patient was lethargic, moving all four extremities, and withdrawing from painful stimulation. At the same time, the patient was placed on a cardiorespiratory monitor, and initial vital signs showed a blood pressure of 127/67 mmHg, heart rate of 142 beats per minute, respiratory rate of 6 breaths per minute, a saturation of 94% on 15 L non-rebreather mask that was briefly placed during the initial evaluation, and a temperature of 37°C. BVM was resumed once severe bradypnea was noted.

At first, the patient was noted to have bilateral jerking movements, and there was a concern for seizure activity. Point-of-care glucose was assessed and returned at 216 mg/dL. Lorazepam (1 mg) was given to the patient via obtained intravenous (IV) access. The patient had no symptomatic relief and remained somnolent with bradypnea and bilateral jerking movements. On further physical examination, miotic pupils were noted bilaterally. At that point, the mother of the patient arrived at the bedside and stated that the patient had been playing earlier in the day near the clothing of the biological father, who is a known drug user. The mother also stated that the weight of the child was 8 kg at her last pediatrician visit. A presumed diagnosis of opioid overdose was made, and the patient was given 0.8 mg of intravenous naloxone. Concurrently, anesthesia was at the bedside and prepared for possible intubation. After the naloxone was administered, the patient immediately was able to sit up and had a respiratory rate of 24 breaths per minute. The patient was suctioned and placed on her side and was protecting her own airway with preserved cough and gag reflexes. Laboratory studies were sent, including a urine drug screen, which includes opiates, barbiturates, benzodiazepines, cocaine, and methadone with the addition of qualitative tetrahydrocannabinol (THC) and fentanyl urine tests.

**Figure 1:**
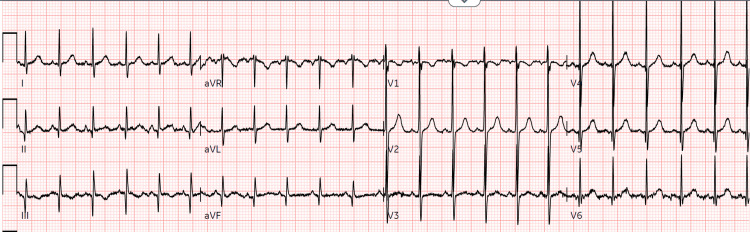
ECG: normal sinus rhythm at 134 beats per minute ECG: electrocardiogram

**Figure 2:**
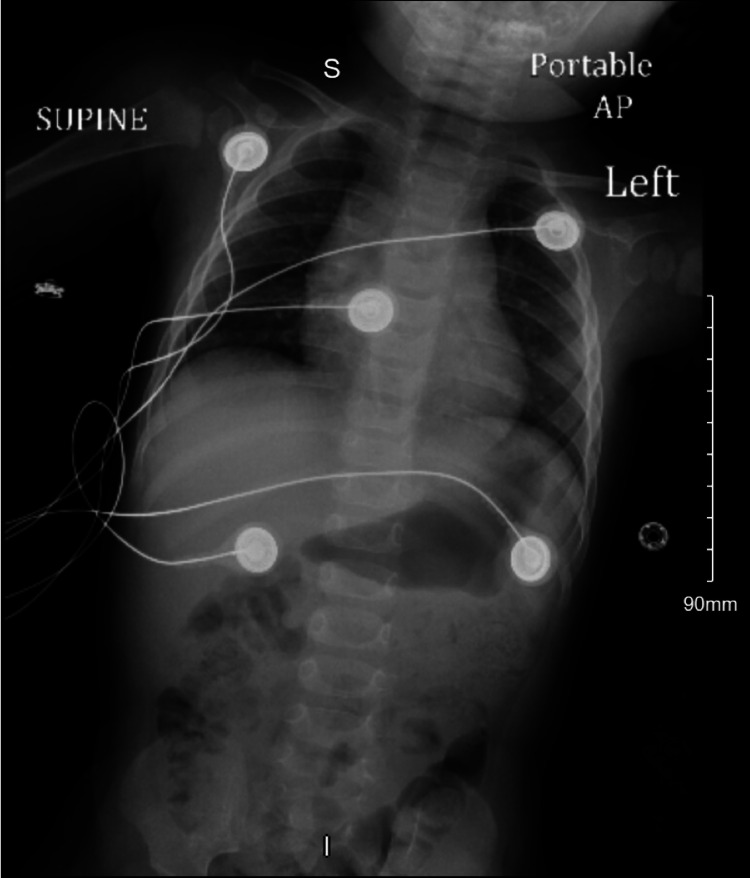
Chest X-ray demonstrating no acute pathology

**Figure 3:**
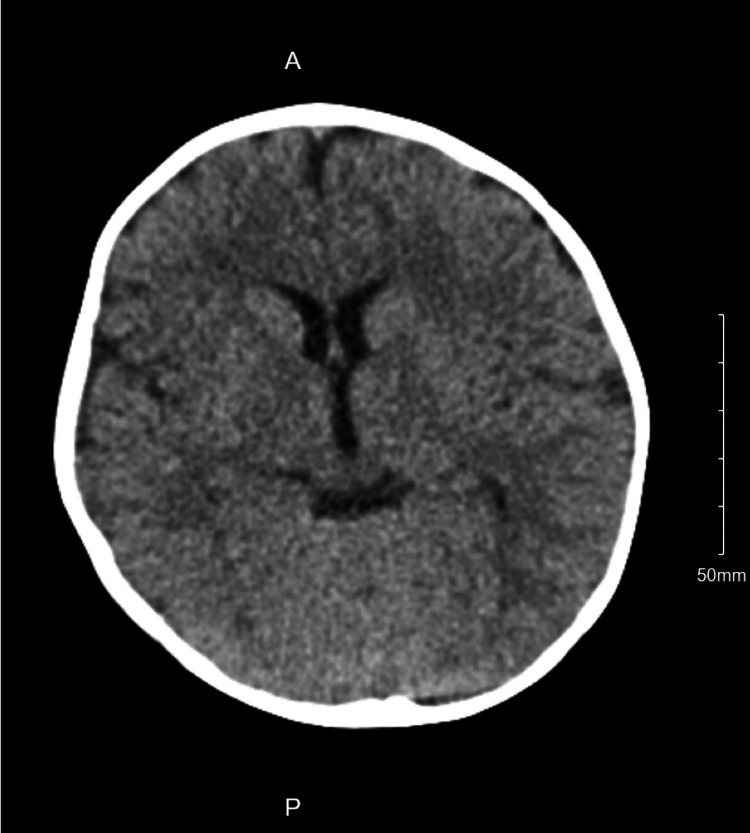
CT scan of the head showing no acute pathology CT: computed tomography

An initial electrocardiogram (ECG) and chest X-ray (CXR) were obtained and are shown in Figure 1 and Figure 2 and aided in excluding other etiologies. ECG showed normal sinus rhythm at a rate of 134 beats per minute. CXR showed no acute pathology. A computed tomography (CT) scan of the head was also obtained (Figure 3) to rule out any intracranial pathology and showed no abnormalities. A standard urine drug screen returned positive for cocaine and opiates. Other laboratory studies that were significant included a white blood cell count of 15.59 × 10^3^/mcL, with no elevated neutrophils and no bands. Urinalysis was unremarkable. Acetaminophen, salicylate, THC, and ethanol levels were negative. The patient was further monitored in the emergency department. She continued to have an intact airway, which she protected with normal vitals. The patient had no further episodes of emesis. Transport was arranged to a nearby hospital for pediatric intensive care. The unfortunate cause of possible intentional poisoning of the child was also considered, and child protective services were contacted. The most likely cause was accidental ingestion of fentanyl, as the child was in the same house as her biological father who was a known drug user. The patient was stable when emergency medical services (EMS) arrived for the transfer approximately 60 minutes later; however, during transport, the providers noted bradypnea once again and administered another dose of naloxone. Therefore a total of two doses and 1.6 mg of naloxone was administered. A few days later, fentanyl, a send-out-of-hospital test performed by gas chromatography-mass spectrometry, came back positive as well. Blood and urine cultures were negative.

## Discussion

There were a total of 23,029 pediatric ingestions reported to poison control from 2020 to August 2022 for children under the age of one [4]. Of these cases, there was only one case of fentanyl and two cases of heroin ingestion that were reported [4]. Fentanyl ingestion in pediatrics carries an extremely high mortality risk, estimated at a rate of nearly 50% [5]. Therefore, the quick diagnosis and IV administration of naloxone to our patient undoubtedly significantly improved her outcome, specifically preventing her from being intubated and placed on mechanical ventilation.

There was consideration for the possible route of exposure for the nine-month-old infant. The patient was still within the age group of breastfeeding, and the mother admitted to breastfeeding. However, studies have shown that only small percentages of fentanyl are transferred through breast milk [7,8]. Some studies have shown as little as 0.033% of the fentanyl consumed by the mother transfers to the breast milk of the infant [7]. However, these studies were performed on patients in the labor and delivery unit who were not consuming illicit fentanyl obtained on the street. However, the number of accidental pediatric opioid ingestions has increased in parallel with the increase of opioid prescriptions of adults, presumably many of them being parents [9].

The use of naloxone and adequate ventilation with a BVM, which is currently recommended as an early intervention for opioid overdose in the American Heart Association pediatric advanced life support guidelines, likely saved the patient’s life [10]. However, the provider must consider possible side effects whenever a drug is administered. In the world of adult emergency medicine, the side effect of naloxone-induced noncardiogenic pulmonary edema is a well-known risk that providers fear upon opiate or opioid reversal. However, in the pediatric age group, there have only been a few cases reported, indicating that they may be at lower risk [11-13].

On that note, this specific case updated the institutional policy at the hospital, and the pediatric resuscitation room is now equipped with intranasal naloxone. Intranasal naloxone has been shown to work in pediatric patients, and peak concentration is reached within 20 minutes in more than half of patients per one prior study [14]. However, there are currently no pediatric intranasal naloxone kits available on the formulary for the hospital; therefore, the pediatric resuscitation room was equipped with intranasal naloxone kits for adults, which are dosed at 4 mg. Intranasal naloxone is approved by the United States Food and Drug Administration at this current dose for pediatrics [15]. Although this is an increased dose than the 0.1 mg/kg suggested by the Committee of Pediatrics in 1990 [16], the low risk of noncardiogenic pulmonary edema or other side effects of naloxone is outweighed by the benefit of resuscitation. It is noted that parenteral, intravenous, intramuscular, or intraosseous naloxone is noted to act more expeditiously than intranasal; however, we have the intranasal form as an immediate option for cases where care or access may be delayed [17].

Our case had certain limitations, including not evaluating for more coingestants and not testing the mother for drug use. This case shows the importance of keeping ingestion on the differential even with younger pediatric age groups and the importance of recognizing an opioid toxidrome even in these extremes of age. This prompted an alteration in our policy at our institution whereby we now include intranasal naloxone in the pediatric resuscitation room. Our hope is that this case will promote a positive shift at other institutions as well.

## Conclusions

Our patient presented cyanotic and bradypneic, putting opioid toxidrome high on the differential. Pediatric ingestions, while more commonly thought of in the toddler age group of 1-3 years old, should be considered in the entire pediatric population. Out of all ingestions, the “one pill can kill” tends to be the most concerning to emergency medicine providers, but illicit drug exposure must not be overlooked, especially when presenting with respiratory failure.
